# The Dietetic Inventiveness of Hunger

**Published:** 1860-07

**Authors:** 


*The Dietetic Inventiveness of Hunger.*—Captain Marcy, in his interesting work, "The Prairie Traveller," supplied several facts not without importance in a physiological and medical aspect. Indeed, under the hardships and privations which fall to the lot of travellers beyond the tracks of civilized life, man and the inferior animals are frequently made the subjects of experiments, which cannot be instituted in our schools and hospitals. Captain Marcy thus describes what befell his party when passing over the Rocky Mountains during the winter of 1857–8;—"Our supplies of provisions were entirely consumed eighteen days before reaching the settlements in New Mexico, and we were obliged to resort to a variety of expedients to supply the deficiency. Our poor mules were fast falling and dropping down from exhaustion in the deep snows, and our only dependence for the means of sustaining life was upon these starved animals as they became unserviceable. We had no salt, sugar, coffee, or tobacco, which, at a time when men are performing the severest labor that the human system is capable of enduring, was a great privation. In this destitute condition we found a substitute for tobacco in the bark of the red willow, which grows upon many of the mountain streams in that vicinity. The outer bark is first removed with a knife, after which the inner bark is scraped up into ridges around the sticks, and held into the fire until it is thoroughly roasted, when it is taken off the stick, pulverized in the hand, and is ready for smoking. It has the narcotic properties of tobacco, and is quite agreeable to the taste and smell. The sumach leaf is also used by the Indians in the same way, and has a similar taste to the willow bark. A decoction of the dried wild or horse mint, which we found abundant under the snow, was quite palatable, and answered instead of coffee. It dries up in that climate, but does not lose its flavor. We suffered greatly from the want of salt; but by burning the outside of our mule-steaks, and sprinkling a little gun-powder upon thom, it did not require a very extensive stretch of the imagination to fancy the presence of both salt and pepper. (There can be no doubt that the nitre and charcoal would to a great extent supply the want of salt. Soldiers and huntsmen have long resorted to this use of gun-powder under emergencies.) We tried the flesh of horse, colt, and mule, all of which were in a starved condition, and of course not very tender, juicy, or nutritious. We consumed the enormous amount of from five to six pound of this meat per man daily; but continued to grow weak and thin, until, at the end of twelve days, we were able to perform but little labor, and were continually craving for fat meat."

